# AI-driven generalized polynomial transformation models for unsupervised fundus image registration

**DOI:** 10.3389/fmed.2024.1421439

**Published:** 2024-07-16

**Authors:** Xu Chen, Xiaochen Fan, Yanda Meng, Yalin Zheng

**Affiliations:** ^1^Department of Medicine, University of Cambridge, Cambridge, United Kingdom; ^2^Institute of Ophthalmology, University College London, London, United Kingdom; ^3^Department of Computer Science, University of Exeter, Exeter, United Kingdom; ^4^Department of Eye and Vision Sciences, University of Liverpool, Liverpool, United Kingdom; ^5^Liverpool Centre for Cardiovascular Science, Liverpool, United Kingdom

**Keywords:** image registration, unsupervised learning, polynomial transformation, foundational model, color fundus photography

## Abstract

We introduce a novel AI-driven approach to unsupervised fundus image registration utilizing our Generalized Polynomial Transformation (GPT) model. Through the GPT, we establish a foundational model capable of simulating diverse polynomial transformations, trained on a large synthetic dataset to encompass a broad range of transformation scenarios. Additionally, our hybrid pre-processing strategy aims to streamline the learning process by offering model-focused input. We evaluated our model's effectiveness on the publicly available AREDS dataset by using standard metrics such as image-level and parameter-level analyzes. Linear regression analysis reveals an average Pearson correlation coefficient (R) of 0.9876 across all quadratic transformation parameters. Image-level evaluation, comprising qualitative and quantitative analyzes, showcases significant improvements in Structural Similarity Index (SSIM) and Normalized Cross Correlation (NCC) scores, indicating its robust performance. Notably, precise matching of the optic disc and vessel locations with minimal global distortion are observed. These findings underscore the potential of GPT-based approaches in image registration methodologies, promising advancements in diagnosis, treatment planning, and disease monitoring in ophthalmology and beyond.

## 1 Introduction

Image registration is an essential process in vision applications where multiple images obtained from different viewpoints or spaces, are aligned. In medical imaging, this technique holds significant importance, enabling the comparison and analysis of images to gain insights into structural changes, disease progression, and treatment efficacy. The primary objective of image registration is to align two images, denoted as a fixed image (target) *F* and a moving image (source) *M*, by establishing spatial correspondence within a shared coordinate system. In a simpler term, assuming *x* and *y* represent the column and row indices, image registration involves mapping a position (x, y) from *M* to a new warped/aligned image *W* at position (*u*(*x, y*), *v*(*x, y*)), where *u* and *v* denote different types of transformation functions. Image registration encompasses linear and non-linear transformations. Linear transformations involve global geometric adjustment of the moving image, while non-linear transformations allow for local or regional deformations to the moving image. Linear transformations often serve as the prerequisite step for non-linear registration techniques by addressing global distortions from differing viewpoints, making them an essential component in the image registration pipeline. The most basic linear transformation type for image registration is translation, wherein *u* and *v* can be expressed in Equation (1):


(1) $$\begin{array}{l} u=x+t_{x}\text{ and }v=y+t_{y} \end{array}$$

Here, *t*_*x*_ and *t*_*y*_ represent the translation lengths along the respective axes. Affine transformation is a common linear technique employed in image registration to address distortions arising from non-ideal camera angles. Typically, an affine transformation encompasses four fundamental operations: rotation, translation, scaling, and shearing. The expressions for *u* and *v* in the context of affine transformation are given (see Equation 2):


(2) $$\begin{array}{l} u=a_{00}x+a_{01}y+t_{x}\text{ and }v=a_{10}x+a_{11}y+t_{y} \end{array}$$

where, *a*_00_, *a*_01_, *a*_10_, *a*_11_, *t*_*x*_ and *t*_*y*_ are the transformation parameters. The planetary of surfaces, parallelism and angles between lines are all preserved in affine transformation. Furthermore, projective transformation is a type of geometric transformation that maps points in one plane to another plane using a projective matrix. It involves transforming points in a two-dimensional space, such as an image, to another two-dimensional space, allowing for changes in perspective, rotation, skewing, and other distortions. The expressions for *u* and *v* in the context of projective transformation is given in Equation 3:


(3) $$\begin{array}{l} u=\frac{b_{00}x+b_{01}y+b_{02}}{b_{03}x+b_{04}y+c},\;v=\frac{b_{10}x+b_{11}y+b_{12}}{b_{13}x+b_{14}y+c} \end{array}$$

where *b*_00_-*b*_14_ are the projective transformation parameters; *c* represents the coefficient associated with the z-coordinate in homogeneous coordinates. It is commonly referred to as the projective invariant and is used to represent the translation component of the transformation. Projective transformations are frequently employed in retinal image registration and geometric correction (1, 2). Retinal image registration is crucial in the diagnosis of eye diseases as it enables the accurate assessment of disease-related features and progression. Fundus imaging, including color fundus photography, optical coherence tomography (OCT), fluorescein angiography and other advanced imaging modalities, provides essential visual information for the diagnosis and management of retinal diseases and systemic diseases (3–5). The registration of fundus images allows for the alignment and comparison of images over time, facilitating the identification of changes in related features such as drusen, geographic atrophy (GA), and choroidal neovascularization (CNV) (6, 7). Fundus image registration is particularly important in the context of multi-modal imaging, where the integration of different imaging modalities such as OCT and fluorescein angiography enhances the comprehensive assessment (4, 5). By registering fundus images with other imaging modalities, clinicians can obtain a more comprehensive understanding of the structural and functional changes, leading to improved diagnostic accuracy and prognostic evaluation (8, 9). Moreover, the application of advanced technologies such as deep learning has shown promise in leveraging fundus image registration for the differential diagnosis, as well as for the automated segmentation of related lesions such as GA (10–12). These technological advancements enable the precise analysis of fundus images, contributing to the development of prognostic biomarkers and the prediction of disease progression (13). Deep learning-based image registration has emerged as a promising approach, offering solutions for linear transformations using convolutional neural networks (CNNs). The Spatial Transformer Network (STN) was among the pioneering CNN-based methods, focusing on learning two-dimensional affine transformations for distorted MNIST digit classification through supervised learning. Miao et al. (14) introduced a supervised CNN approach to regress three-dimensional transformation matrices for affine registration of X-ray images, utilizing synthesized transformation parameters as ground truth. However, the reliance on labeled ground truth for supervised methods can be limiting, prompting the development of unsupervised models that do not require transformation ground truth. De Vos et al. (15) proposed an unsupervised Deep Learning Image Registration (DLIR) framework, enabling joint affine and nonlinear registration without the need for labeled ground truth. The affine transformation framework within DLIR employs a multi-stage approach tailored for multi-temporal image registration. Additionally, Chen et al. (16) proposed an unsupervised CNN approach focused on explicitly learning specific geometric transformation parameters such as translations, rotations, scaling, and shearing. Unlike traditional methods that regress affine transformation matrices, this approach targets individual transformation parameters, offering a tailored solution for affine registration tasks in multi-modality image registration scenarios.

Current limitations in deep learning-based models for image registration are: (1) While much attention has been devoted to affine transformation for linear registration in deep learning-based models, real-world scenarios often involve more complex distortions that may not be adequately addressed by affine transformation alone. Powerful and complex linear registration techniques, such as projective transformation or polynomial transformation, offer additional flexibility in capturing the intricacies of image distortions. Affine transformations, while effective for linear registration tasks, have limitations in capturing non-linear distortions or irregular deformations present in many medical imaging applications. By incorporating projective or polynomial transformations, which allow for non-linear and higher-order transformations, these techniques can better model the intricate variations and deformations encountered in medical images. This enhanced flexibility enables more accurate alignment and registration of images, leading to improved diagnostic and analytical outcomes. However, the exploration of these techniques in the context of deep learning-based image registration remains limited. (2) Lack of generalized models for image transformation: One significant limitation in the realm of deep learning-based image registration lies in the absence of generalized models capable of learning image transformations universally. Many existing models are meticulously designed for specific images and modalities, hindering their adaptability to a broader range of scenarios. This limitation restricts the scalability of these models, making them less effective in scenarios where a diverse set of images or modalities is encountered. Consequently, the field faces challenges in achieving a more comprehensive and generalized approach to image transformation learning and expansion.

## 2 Methods

**Figure 1:**
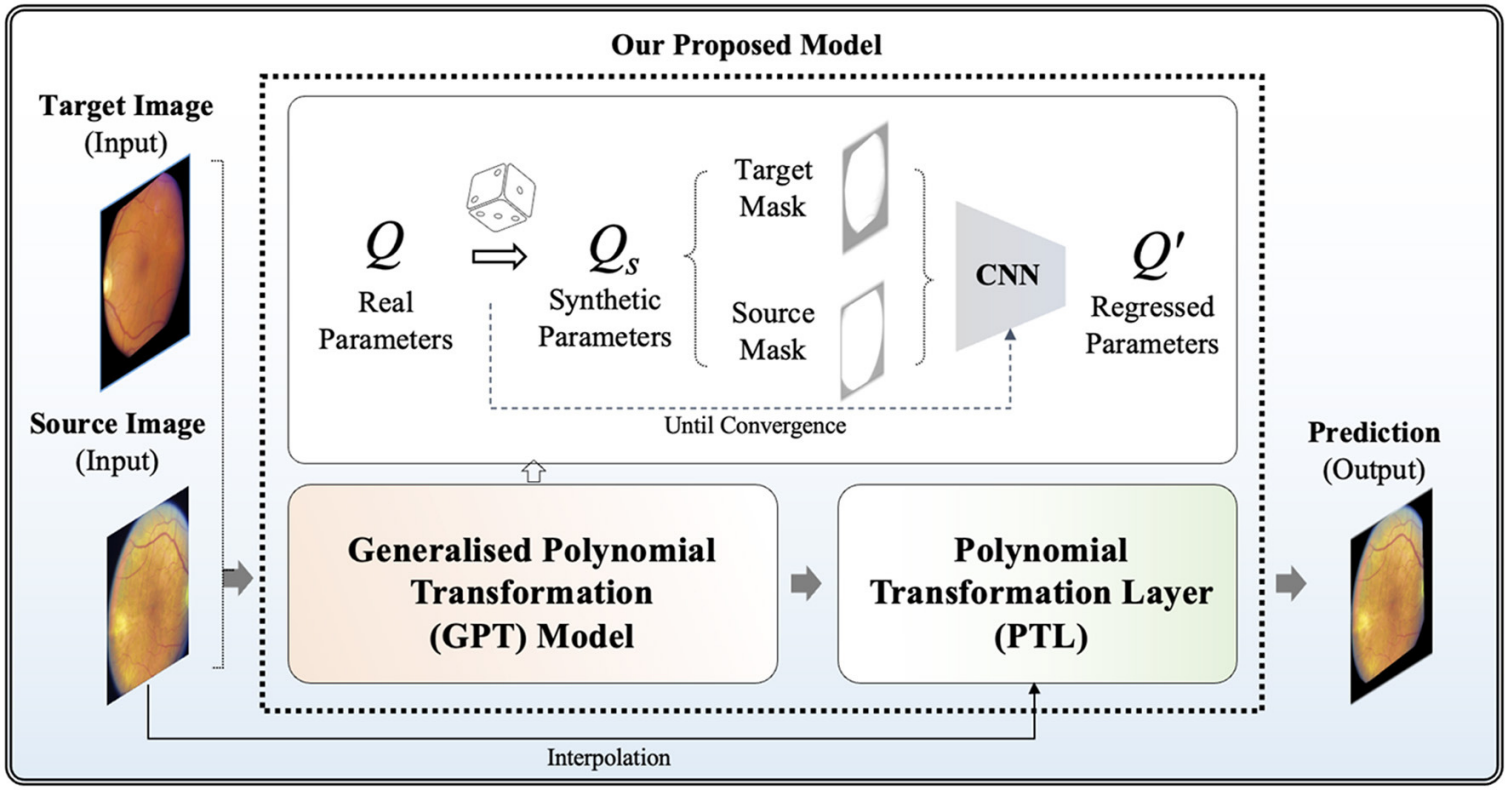
Overview of our proposed model for unsupervised polynomial image registration.

Addressing the constraints observed in existing deep learning-based fundus image registration models, we proposed a generalized model that introduces an unsupervised approach tailored specifically for quadratic transformations, the second degree of polynomial transformation. Polynomial transformation is a process in which the input features are transformed by using a polynomial function of a certain degree. The goal of polynomial transformation is to capture more complex relationships between the features and the target variable than a simple linear model would. It can be useful when the relationship between variables is curvilinear rather than linear. However, higher-degree polynomials can also lead to over-fitting, so the degree of the polynomial should be chosen carefully based on the characteristics of the data. Mathematically, the *u* and *v* for polynomial transformation can be defined in Equation 4:


(4) $$\begin{array}{l} u=\overset{p}{\underset{d=0}{\sum }}\overset{p-i}{\underset{d=0}{\sum }}a_{d}x^{d}y^{d}\text{ and }v=\overset{p}{\underset{d=0}{\sum }}\overset{p-i}{\underset{d=0}{\sum }}b_{d}x^{d}y^{d} \end{array}$$

where *p* is the degree of polynomial and *a*_*d*_, *b*_*d*_, are the transformation parameters. These transformations include linear (*p* = 1), quadratic (*p* = 2), cubic (*p* = 3), bi-quadratic (*p* = 4) and quintic (*p* = 5) ones as special cases. For this work, quadratic (*p* = 2) transformation is used and can be expressed as:


(5) $$\begin{array}{l} \left[\begin{array}{c} u\\ v \end{array}\right]=Q{\left[\begin{array}{cccccc} x^{2} & y^{2} & xy & x & y & 1 \end{array}\right]}^{T}\\ \;=\left[\begin{array}{cccccc} q_{00} & q_{01} & q_{02} & q_{03} & q_{04} & q_{05}\\ q_{10} & q_{11} & q_{12} & q_{13} & q_{14} & q_{15} \end{array}\right]{\left[\begin{array}{cccccc} x^{2} & y^{2} & xy & x & y & 1 \end{array}\right]}^{T} \end{array}$$

where **Q** is the quadratic transformation matrix. For image registration tasks, quadratic transformation can be formulated as an energy minimization problem (see Equation 6):


(6) $$Q^{*}=\underset{Q}{\text{arg max}}\left\{Q|S(F,QM))\right\}$$

where S is the metrics to measure the similarity between a fixed image *F* and the warped image **Q***M*. Our model aims to optimize each individual transformation parameter *q*_00_ - *q*_15_, instead of directly optimizing the transformation matrix **Q**. In the following sections, we provide more details of our framework, highlighting its two distinct features: the Generalized Polynomial Transformation (GPT) model and an unsupervised GPT-based transformation model specifically tailored for fundus image registration. The overview of our proposed model is represented in Figure 1.

Firstly, we propose the GPT model, serving as a foundational model to emulate diverse polynomial transformations. To construct a synthetic dataset to acquire knowledge of the quadratic transformation, we randomly selected each *q* parameter from the raw **Q** and then generated a synthetically wrapped image by the new **Q**_*s*_ matrix according to Equation (5). More specifically, each *q*∈*Q*_*s*_ is derived from the distribution of the corresponding *q*∈*Q*. For example, *q*_1_5 ranges from 651.0 to –278.0 across the non-hold out testing set, so the new *q*_1_5 is assigned a random value within this range. Given unlimited combination, we developed an "on-the-fly" synthetic dataset generation approach during training steps, continuously generating synthetic data until the model achieved full convergence. To achieve full convergence, the "on-the-fly" synthetic data generator continuously produces random parameters for each epoch. This process involves generating a diverse set of synthetic image pairs by applying various quadratic transformations. The synthetic data generator operates iteratively, introducing new transformation parameters in each epoch to ensure that the model is exposed to a wide range of transformation scenarios. The training continues until the evaluation accuracy on the validation dataset stabilizes, indicating that the model has effectively learned the transformation characteristics and can generalize unseen data. This dynamic approach helps prevent over-fitting and ensures robust performance by leveraging an ever-expanding dataset that reflects the complex nature of real-world transformations. This step offers the advantage of automatically generating ground truth data without the need for manual annotations. It enables GPT to investigate a broad spectrum of polynomial transformation scenarios, encompassing nearly all possible transformation combinations. This strategy allows its convolutional neurons to be activated appropriately when capturing relevant features for geometric transformation. Without employing the "on-the-fly" synthetic dataset generation approach, the convolutional neurons in the model might be influenced by potential biases arising from a limited number of training samples. This could lead to sub-optimal learning outcomes and reduced model generalization ability, as the network may not adequately capture the full variability and complexity of the transformation space in **Q**. By continuously generating synthetic data on the fly, the model receives a diverse and extensive training dataset, mitigating the risk of over-fitting and enhancing its ability to learn robust representations of quadratic transformations.

In this study, the GPT model is trained using binary masks extracted from fundus images, where non-black areas are encoded as 1, and black areas are assigned a value of 0. This strategic approach enables the model to focus on capturing the global features of transformation between images while filtering out irrelevant local features such as vessels. By prioritizing the essential structural elements of the images, the GPT model can effectively learn and reproduce accurate geometric transformations, leading to improved image registration performance. A well-tuned GPT model can be extended as a generalized model across various imaging modalities where polynomial transformations are required, providing a versatile solution for image registration tasks.

The development of our GPT model is based on the EfficientNetV2 architecture (17), which is chosen for its well-established balance between model complexity and computational efficiency, rendering it ideal for training on a large synthetic dataset. The global max pooling layer was introduced in GPT because it can enhance the GPT model's ability to focus on essential features contributing to overall image transformation. The output layer is a linear activation function, facilitating the generation of regressed parameters for the randomly polynomial-transformed image. To guide the training process effectively, we propose a hybrid loss function in Equation 7, denoted as $L_{hybrid}$, which combines Mean Squared Logarithmic Error (MSLE) (Equation 8) and Cosine Similarity (CoS) (Equation 9). In which, ω represents the weighting factor for balancing between two terms. Specifically, in our implementation, we set ω to 0.5 to ensure equal contribution from both terms.


(7) $${ℒ}_{hybird}(Q,Q^{\prime })=\omega MSLE+(1-\omega )(1-CoS)$$

where,


(8) $$CoS(Q,Q^{\prime })=\frac{Q\cdot Q^{\prime }}{\left\|Q\right\|\times \left\|Q^{\prime }\right\|}=\frac{\sum \left(Q\times Q^{\prime }\right)}{\sqrt{\sum Q^{2}}\times \sqrt{\sum {Q^{\prime }}^{2}}}$$

(9) $$MSLE(Q,Q^{\prime })=\frac{1}{N}\sum_{i=0}^{N}{\left[\text{ log }(Q_{i}+1)-\text{ log }(Q^{\prime }{\;}_{i}+1)\right]}^{2}$$

Given the diverse ranges of parameters (*N* = 16) within **Q**, MSLE serves as a robust loss measure. By utilizing a logarithmic scale, MSLE effectively addresses large outliers, treating them comparably to smaller deviations. This feature is particularly advantageous for ensuring model balance, especially when striving for uniform percentage errors across **Q**. To address negative values of parameters within **Q**, CoS evaluates the directional consistency between vectors of **Q** and **Q**′, offering significant utility when handling transformations that incorporate negative values. Our $L_{hybrid}$ loss functions fortify the GPT model, empowering it to adeptly capture and mimic diverse polynomial transformations with resilience and efficacy.

Note that the pre-trained GPT model cannot be directly applied to real fundus image pairs because it was tuned using binary masks and is not trained with any local features such as vessels and the optic disc. In the methodology of our model for unsupervised fundus image registration, the pre-trained GPT model is severed as the foundation, namely pre-trained weights, leveraging its capabilities in capturing diverse polynomial transformations to train a new tailored model for fundus image registration. In which, we proposed a new Polynomial Transformation Layer (PTL) to warp *M* by the regressed transformations **Q**′. In PTL, interpolations of **Q**′*M* can be formulated in Equation (10) according to Equation (5):


(10) $$\begin{array}{l} u=q_{00}x^{2}+q_{01}y^{2}+q_{02}xy+q_{03}x+q_{04}y+q_{05},\\ \;v=q_{10}x^{2}+q_{11}y^{2}+q_{12}xy+q_{13}x+q_{14}y+q_{15} \end{array}$$

The objective is to maximize the similarity between the transformed image **Q**′*M* and the target image *F*, facilitating unsupervised image registration as the model encounters real transformed images. The loss function $L_{unsupervised}$ (see Equation 11) is based on Normalized Cross Correlation (NCC) by measuring the correlation between corresponding pixel values.


(11) $$\begin{array}{l} \;{ℒ}_{unsupervised}(F,Q^{\prime }M)\\ =1-\frac{\sum_{x,y}(F(x,y)-\bar{F})(Q^{\prime }M(x,y)-\bar{Q^{\prime }}M)}{\sqrt{\sum_{x,y}(F(x,y)-\bar{F}{)}^{2}\sum_{x,y}(Q^{\prime }M(x,y)-\bar{M}{)}^{2}}} \end{array}$$

## 3 Experiments

In this section, we detail experiments conducted to validate our GPT-based model for unsupervised fundus image registration. Through a series of experiments and analyzes, we aim to assess the model's ability to accurately align fundus images without the need for ground truth transformation parameters. By detailing the experimental methodology, dataset characteristics, evaluation metrics, and results, we provide insights into the robustness and reliability of our proposed approach in the context of ophthalmic imaging and clinical practice.

### 3.1 Dataset

Our methodology is applied to a longitudinal dataset comprising color fundus images from the AREDS study (18), captured using the Zeiss FF-series 30-degree fundus camera at baseline, 2-year, and subsequently annually (19). Extracting longitudinal color fundus images from 4,903 eyes (involving 2,702 participants) sourced from the AREDS study, each patient underwent a minimum of three follow-up visits after the baseline examination. Categorizing the fundus images into non-advanced (early/intermediate stage) and advanced (late stage) AMD, with advanced AMD characterized by the presence of drusen or geographic atrophy, the dataset is publicly available upon request from the database of Genotypes and Phenotypes (dbGaP; accession: phs000001.v3.p1). All analyzes adhere to the approved research use statement.

### 3.2 Pre-processing

In our approach to unsupervised fundus image registration, we recognize the significance of targeted pre-processing to enhance the model's focus on crucial features. We introduced a hybrid pre-processing approach incorporating both Contrast Limited Adaptive Histogram Equalization (CLAHE) (20) and bilateral filter (21).

**Figure 2:**
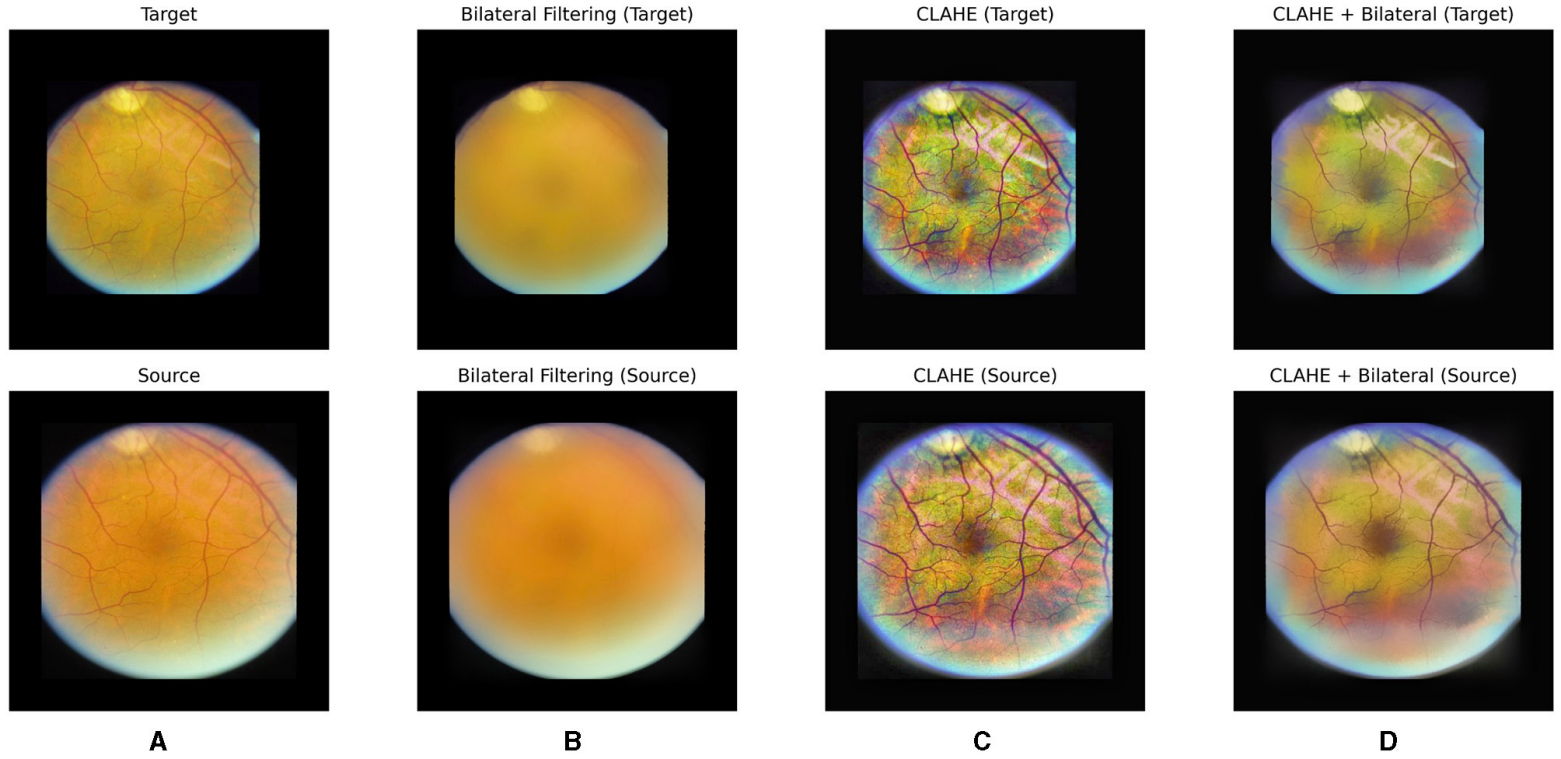
Our hybrid pre-processing strategy incorporates both Contrast Limited Adaptive Histogram Equalization (CLAHE) and bilateral filter. From left to right, raw images **(A)**, post-bilateral filtering **(B)**, post-CLAHE **(C)** and images after hybrid pre-processing for a pair of fundus images are displayed, respectively.

CLAHE is a preprocessing technique particularly beneficial for enhancing contrast and improving image quality in fundus images. By locally adapting the contrast enhancement process, CLAHE ensures that the contrast improvements are tailored to the specific characteristics of different regions within the image. This helps in bringing out subtle details and structures in fundus images, such as blood vessels and pathological features. Additionally, CLAHE helps in reducing the impact of uneven illumination and varying brightness levels often present in fundus images, thereby aiding in standardizing the image appearance and facilitating more reliable analysis algorithms. However, as observed in Figure 2C, CLAHE may inadvertently over-enhance unnecessary features in fundus images. Consequently, to address this issue and further denoize the images, a bilateral filter was introduced as a subsequent step in the preprocessing pipeline.

A bilateral filter is employed to systematically eliminate irrelevant and non-diagnostic elements from fundus images. The bilateral filter acts as a selective tool, smoothing the images while preserving essential features such as the optic disc and blood vessels. By doing so, we effectively reduce noise and unwanted details, creating a cleaner input for the subsequent learning stages. This refined dataset allows our model to concentrate on the pertinent anatomical structures, namely the optic disc and vessels, optimizing its ability to learn and predict image transformations accurately. The impact of the bilateral filter can be observed in Figures 2B, D, where a bilateral filter is applied to raw images (Figure 2A) and post-CLAHE images (Figure 2C), respectively.

With our hybrid pre-processing strategy, the objective is to optimize the learning process by offering the model a concentrated and pertinent input (see Figure 2D). This approach enhances the model's interpretability and fosters a more efficient understanding of fundus images.

### 3.3 Training

The dataset was partitioned at the patient level, with 60% allocated for training, 20% for validation, and 20% for hold-out testing purposes. Training employed the Adam optimizer, a widely embraced algorithm in deep learning, with an initial learning rate of 0.001, facilitating effective model convergence. Input images were resized to 256x256 pixels, and for normalization, we adopted a scale spanning from –1 to 1 instead of the conventional 0–1 range. This deliberate choice prevents the suppression of convolutional neuron activation in black areas, which often contain relevant features for geometric transformation. To enhance model generalization, we applied data augmentation techniques, including flipping, rotation, random brightness, and random contrast.

Our GPT-based model, constructed upon the pre-trained GPT architecture, served as the foundational framework for image registration. Fine-tuning the training set showcased the model's adaptability in capturing diverse Polynomial Transformations, proving advantageous for aligning fundus images. Transparency in our methodology is maintained by providing access to the code, models, and data employed in this experiment, implemented using TensorFlow (version 2.10). During the training phase, where the regression work involves various parameter ranges, we took into account the potential inefficiency of the last linear layer. To address this, our model regressed on normalized values within the [0, 1] range. This strategic approach facilitated a more effective learning process. Once the model converged, we implemented a scaling process to transform each parameter back to its actual range. This scaling step is particularly crucial for subsequent interpolation work, ensuring that the model's learned parameters align accurately with the original data characteristics. By incorporating this normalization and scaling strategy, our methodology enhances the model's adaptability to diverse parameter ranges and contributes to the precision of the final predictions.

### 3.4 Evaluation metrics

To assess the effectiveness of our model, we employed standard evaluation metrics for image registration at both the image level and parameter level. At the parameter-level, Bland-Altman plots and Pearson correlation coefficients were utilized to evaluate the agreement between predicted and ground truth parameters. Bland-Altman plots visually display the agreement between two quantitative measurements by plotting the difference between the paired measurements against their mean. Additionally, correlation coefficients provide a numerical measure of the strength and direction of the linear relationship between two variables, indicating the degree of agreement between predicted and ground truth parameters. Meanwhile, at the image level, Structural Similarity Index (SSIM) and Normalized Cross Correlation (NCC) were employed. These metrics provided a comprehensive assessment of the overall quality of image alignment by measuring both structural and pixel-wise similarity between the predicted and target images (see Equations 11 and 12).


(12) $$SSIM(I_{t},I_{w})=\frac{\left(2\mu_{I_{t}}\mu_{I_{w}}+C_{1})(2\sigma_{I_{t}}\sigma_{I_{w}}+C_{2}\right)}{\left(\mu_{I_{t}}^{2}+\mu_{I_{w}}^{2}+C_{1})((\sigma_{I_{t}}^{2}+\sigma_{I_{w}}^{2}+C_{2}\right)}$$

## 4 Results

In the results section, we extensively assess the performance of our GPT-based model for unsupervised fundus image registration using the AREDS dataset.

**Figure 3:**
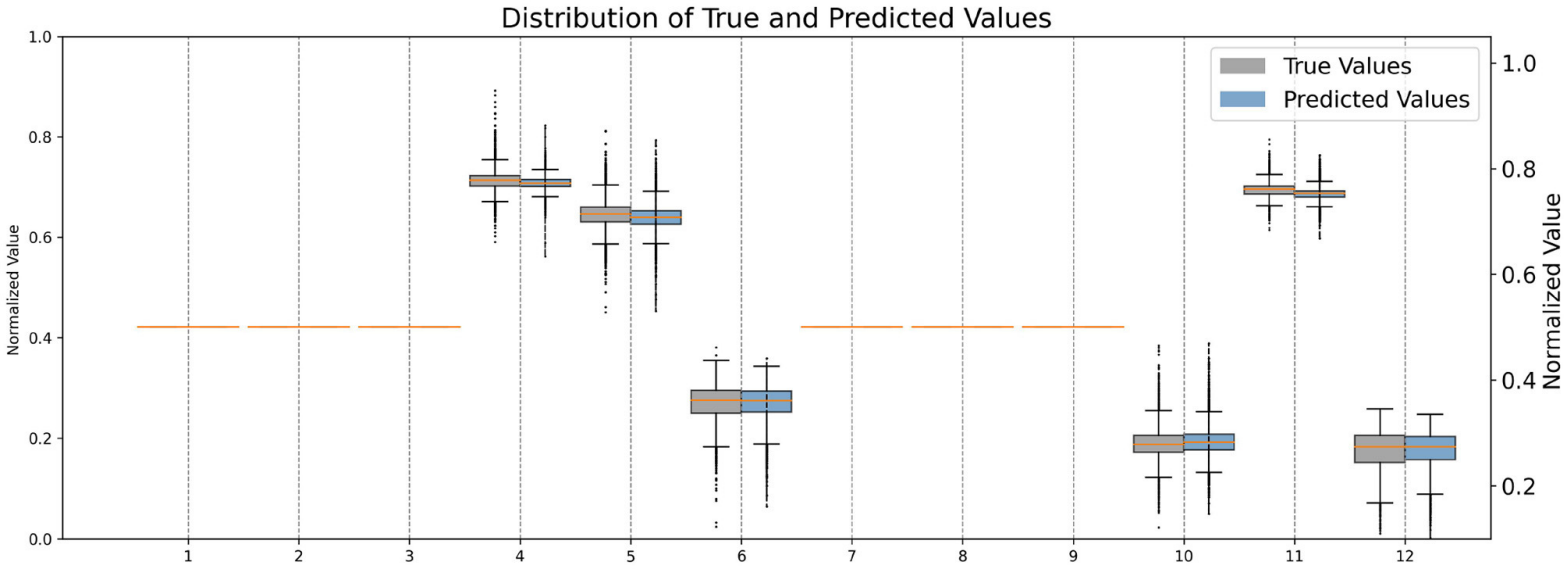
Comparison of normalized distributions of each quadratic transformation parameter.

Firstly, We normalized the predicted and target transformation parameters to a range of 0 to 1 to ensure consistency and comparability between the values. This normalization allows for a standardized scale across all parameters, facilitating easier interpretation and analysis of the data. Additionally, by scaling the parameters to a common range, we mitigate the effects of varying magnitudes and ensure that each parameter contributes proportionally to the overall transformation. Upon comparison of their distributions (see Figure 3), it allows us to visually assess the similarity between the predicted and target parameter distributions, providing insights into the model's performance in capturing the transformation characteristics accurately.

**Figure 4:**
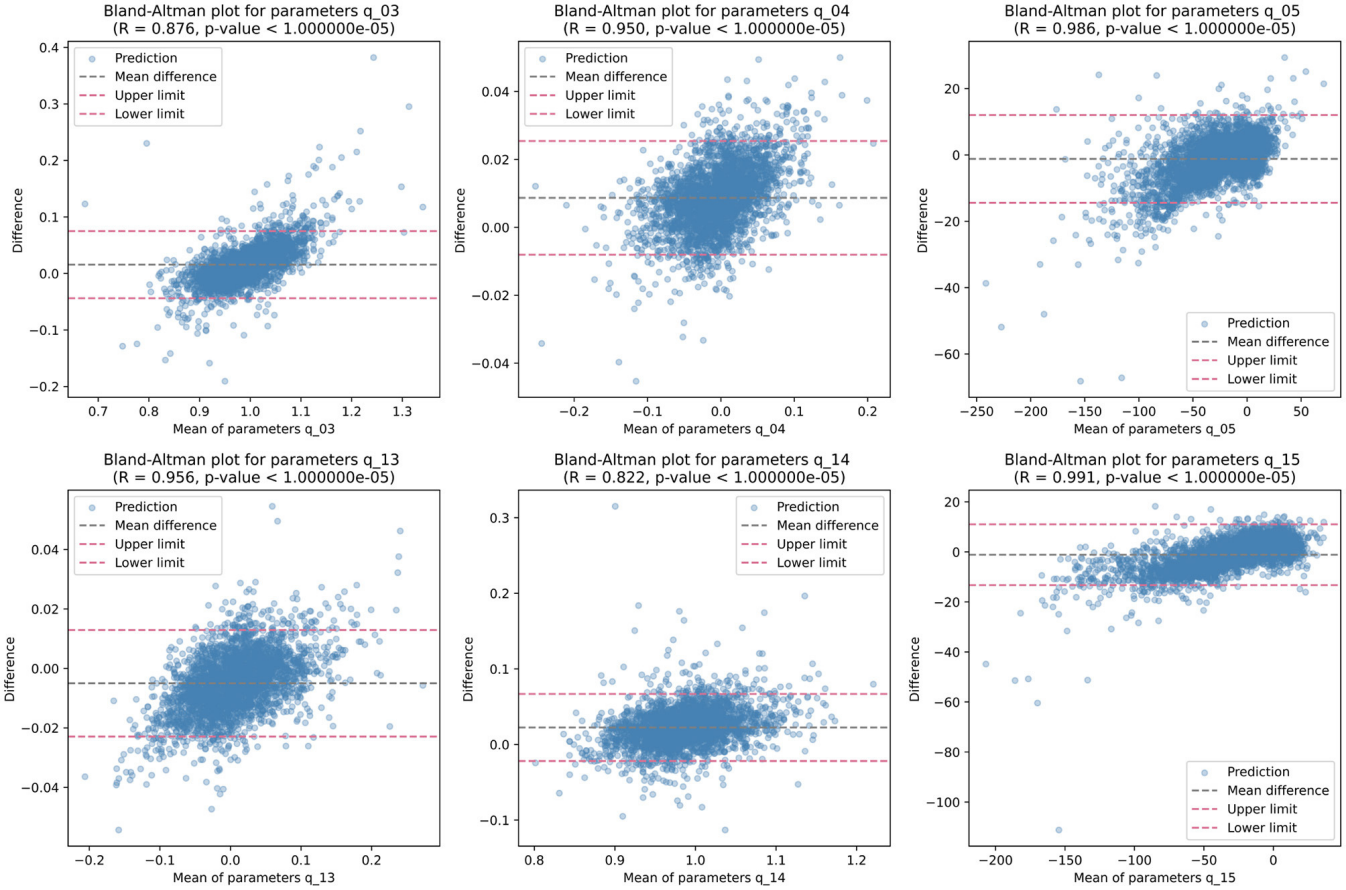
Bland-Altman plots illustrating the distribution of each non-zero quadratic transformation parameter, accompanied by correlation coefficient *R*, *p*-value, mean difference, and upper/lower limits.

Then, we analyzed the non-zero parameters individually, examining their respective distributions and correlations with the target parameters in terms of the correlation coefficient *R*. For each non-zero parameter in **Q**, the correlation coefficient *R* ranges from 0.895 to 0.990, with associated p-values < 0.00001, indicating a strong linear relationship between the predicted and target values. These correlation coefficients signify the degree of agreement between the predicted and target parameters. Figure 4 illustrates the corresponding Bland-Altman plots, showcasing the mean difference and upper/lower limits, providing visual insights into the agreement and potential biases between the predicted and target parameters.

**Figure 5:**
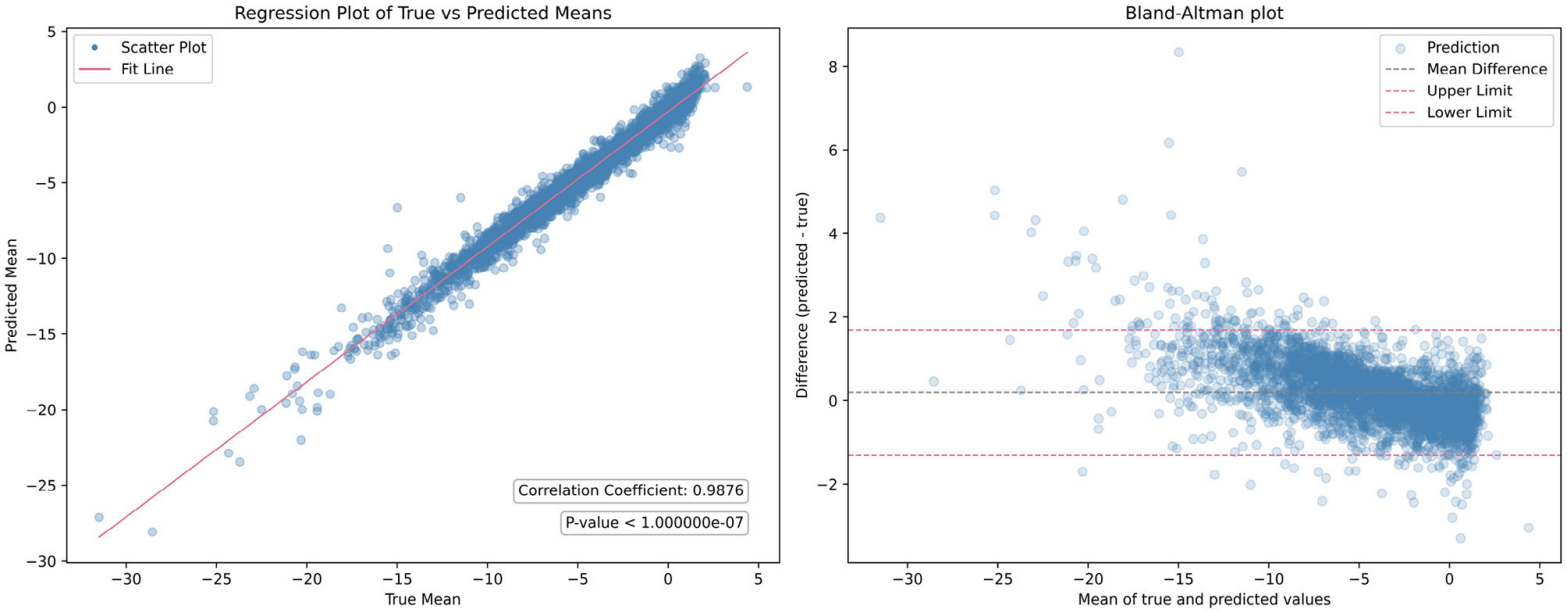
Comparison of the overall mean performance of the generalized polynomial transformation (GPT) model. The **(left figure)** displays the results of linear regression analysis, while the **(right figure)** presents the Bland-Altman plot.

While individual parameters show promising results, the overall mean performance of GPT lacked evaluation. To address this, linear regression analysis was conducted across all quadratic transformation parameters, yielding an average correlation coefficient *R* of 0.9876. Figure 5 illustrates the regression results and corresponding Bland-Altman plot. This high level of correlation underscores the GPT model's ability to accurately predict transformation parameters, demonstrating its efficacy in aligning fundus images without the need for ground truth transformation data.

At the image level evaluation, we conducted both qualitative and quantitative analyzes to comprehensively assess the performance of our model. For the quantitative analysis, we initially evaluated the SSIM and NCC scores before alignment to establish a baseline measurement of similarity between the fixed and moving images prior to any transformations. This baseline provides insights into the initial degree of correspondence before considering the contributions of our models. The SSIM and NCC scores before alignment are 0.6096 and 0.524, respectively. According to Equation (9), the warped images were generated using the transformation parameters (model outputs) based on the corresponding moving images. The SSIM and NCC scores after alignment by our model are 0.8075 and 0.6765, respectively, demonstrating a huge improvement over the baseline. Additionally, the contribution of the pre-processing steps is significant. When these steps are omitted, the SSIM and NCC scores decrease to 0.7649 and 0.6305, respectively.

**Figure 6:**
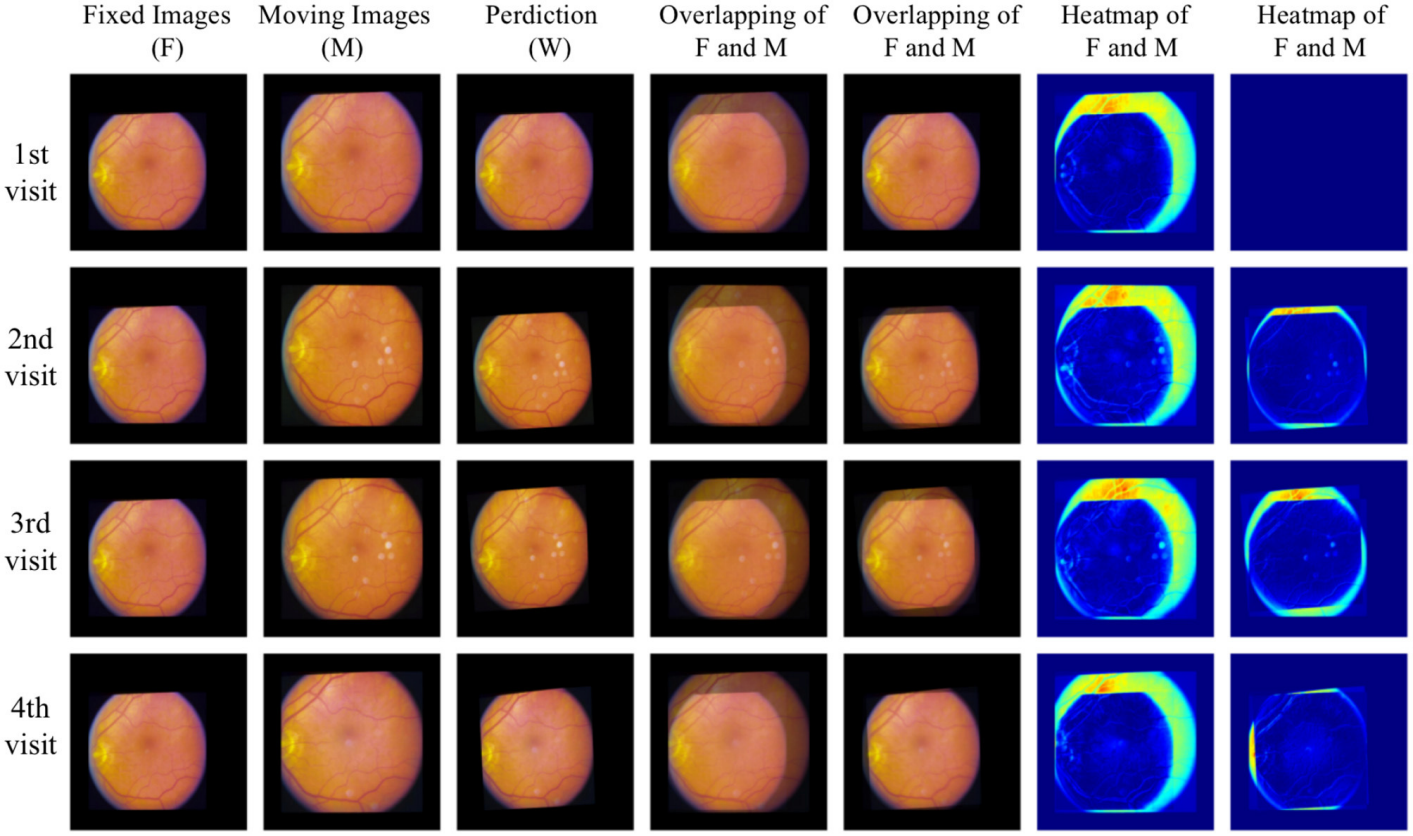
Qualitative analysis with overlapping and heat maps reveals four different visits of one eye.

For qualitative analysis, overlapping and heat maps are employed to visualize differences between images. Figure 6 illustrates the fixed images, moving images, and our warped images in the first three columns across four different visits of one eye. Subsequent columns display the overlap between the fixed images and the moving/warped images, followed by heat maps showcasing differences. Significant differences are observed between fixed images and moving images in the overlapping and heat maps, attributed to variations in image acquisition such as differing camera angles or positions. However, comparing fixed images with our warped images reveals a reduction in differences. Particularly, global distortion is minimized, and the locations of the optic disc and vessels are matched precisely. To the best of our knowledge, our work represents the first unsupervised registration method specifically targeting polynomial transformations. This novel approach sets it apart from the majority of existing models, which predominantly focus on nonlinear or affine transformations. The unique nature of our method introduces challenges in direct comparisons with other models, as their underlying objectives differ significantly.

A limitation of our work is that image intensity-based metrics like SSIM and NCC may not be sufficient for evaluating performance. Variations in illumination or field of view between image pairs can lead to an underestimation of our model's capabilities. It would be beneficial to use ground truth segmentation of the optic disc or vessels for evaluation, as this approach can eliminate irrelevant features from images taken from different viewpoints, providing a more accurate assessment of performance. Moreover, exploring alternative evaluation metrics without segmentation labels that account for these challenges, such as domain-specific similarity measures or perceptual metrics, could provide a more comprehensive assessment of performance in real-world scenarios. In addition to the limitations mentioned, variations in image quality across different datasets or imaging devices may also pose challenges for our model. Addressing these factors and developing robust techniques to handle artifacts could further enhance the reliability and applicability of our approach.

Overall, our GPT model showcases its efficacy in aligning fundus images, presenting a notable advancement in the field of medical image registration. By harnessing the power of deep learning and unsupervised learning techniques, our model achieves remarkable results without relying on ground truth transformation data. This not only streamlines the registration process but also mitigates the need for labor-intensive manual annotation, making the approach more scalable and applicable to large-scale datasets. Furthermore, the versatility of the GPT model allows it to adapt to diverse transformation scenarios, offering a robust solution for aligning fundus images acquired from different sources and modalities.

## 5 Conclusion

Our work presents a novel approach to unsupervised fundus image registration using the GPT model. Through GPT, we introduced a foundational model capable of emulating diverse polynomial transformations, trained on a large synthetic dataset to cover a wide spectrum of transformation scenarios. Additionally, our hybrid pre-processing strategy aims to optimize the learning process by providing the model with focused input. To assess our model's effectiveness, we employed standard evaluation metrics on the publicly available AREDS dataset, including image-level and parameter-level analyzes. Linear regression analysis yielded an average correlation coefficient *R* of 0.9876 across all quadratic transformation parameters. In image-level evaluation, both qualitative and quantitative analyzes were conducted, revealing significant improvements in SSIM (20%) and NCC (15%) scores, indicating robust performance. Particularly noteworthy is the precise matching of optic disc and vessel locations and the minimization of global distortion. Our findings highlight the potential of GPT-based approaches in image registration methodologies, and promising advancements in diagnosis, treatment planning, and disease monitoring in ophthalmology.

## Data availability statement

The original contributions presented in the study are included in the article/supplementary material, further inquiries can be directed to the corresponding author.

## Ethics statement

The studies involving humans were approved by Age-Related Eye Disease Study Research Group (1999). The studies were conducted in accordance with the local legislation and institutional requirements. Written informed consent for participation in this study was provided by the participants' legal guardians/next of kin.

## Author contributions

XC: Conceptualization, Data curation, Investigation, Methodology, Software, Writing – original draft, Writing – review & editing. XF: Formal analysis, Investigation, Validation, Visualization, Writing – original draft, Writing – review & editing. YM: Resources, Writing – review & editing. YZ: Resources, Supervision, Writing – review & editing.
